# News and Notes

**Published:** 1911-10

**Authors:** 


### NEWS *and* NOTES

### REGULAR MEETING OF FULTON COUNTY MEDICAL SOCIETY, SEPTEMBER 21, 1911.

REPORTED BY R. R. DALY, M. D.

Dr. Kime read a paper on "The Watkins-Wortheim Operaton for Cystocele."

Dr. E. C. Davis said that this was the most ingenious method for one of the most difficult gynecologic operations. It yields good results in carefully selected cases operated by a skilled man. The danger attendant upon wounding the bladder and possibly the uterus was great. It should only be used when the patient expected never to have children. He congratulated Dr. Kime on the paper.

Dr. Kime said in closing that the operation, though very difficult and attended with sterilization, was justified by its success.

Dr. Shallenberger read a paper on "Carcinoma of the Uterus and Some of its Problems."

Dr. J. B. Baird, Sr., said that antiseptics and mild astringents make up the best treatment we can have for the inoperable cases. Cauteries are usually of no value. He regretted that the essayist did not go into greater detail as to the early diagnosis of carcinoma of the fundus.

Dr. F. G. Hodgson said he had enjoyed the paper greatly. His experience has been with the use of radium in these cases. This is easily applied in a small tube enclosed in a rubber finger cot and placed within the cervix at night. It is usually left until morning and it frequently relieves the pain. There is a superficial reaction and some healing at the point of the application, but the results are not a cure. Acetone is a new treatment of value and warrants a trial combined with radium.

Dr. Griffin asked the effects of radium on the tissues in these cases. Dr Hodgson said the new tissue is formed as after a mild burn, but is not deep or lasting.

Dr. Thrash said that most of these cases come to us so late that they were nearly dead when seen. Early cases are amenable to treatment. The cylindrical cell type results generally from chronic endometritis and this disease should always be carefully investigated as a possible cause of carcinoma. Curettage detritus should be examined biologically after evry treatment. The disease is curable only when the cancer cells are removed and this can be done when the surgeon sees the case early enough. After metastasis has taken place and the pelvic glands are infected, no cure may be expected.

Dr. Kime said that the paper suggested on point which he has found valuable in his practice; a twenty-minute operation can be done which will give several months of comfort and relief. It is an amputation of the cervix high up with subsequent curettage and actual cautery. It removes the unpleasant and painful symptoms for the time being.

Dr. Shallenberger in closing said that radium would very likely work well with acetone to relieve the pain but its effects would not be lasting.

---

### PROCEEDINGS OF THE AMERICAN PROCTOLOGIC SOCIETY AT LOS ANGELES, CAL., JUNE 26 and 27, 1911.

### EXTRACTS FROM THE REPORT OF PROCTOLOGIC LITERATURE FROM MARCH, 1910 TO MARCH 1911.

By Samuel T. Earle, M. D., of Baltimore, Md.

*(Continued from Last Issue.)*

*Bacteriology and Urinary Findings of Constipation.—By John L. Jelks, M. D., of Memphis, Tenn.*

The author advances no new theories, but expresses his views of the importance of both chemical and microscopical investigation in connection with clinical proctology, and the values of these examinations in cases of atonic constipation.

He refers to the importance of either finding, or eliminating, the presence of intestinal parasites, that are known to produce lesions in the intestinal coats and ports of entry of bacteria or their toxins. He expresses the belief that the destruction wrought to the sub-mucous structures, the infiltration of plastic material and the contracting, distorting, scarred portion of the bowel, as also the consequent destruction of, and interference with the secreting glands, their ducts and the nerve supply may become important factors in the atonic condition of some patients.

The author believes it is important to make microscopic examinations in all cases of this character,—both of the crude and washed specimens, and of the scrapings from the intestinal wall or from any lesion found in it. He also examines the urine chemically and microscopically, believing this important, owing to the relationship and association of diabetes, kidney insufficiency and diseases of the kidney with cases of atonic constipation.

These examinations of the urine aid in determining the proper course of treatment, especially is this true when indicanuria, casts and sometimes traces of albumen, indicate the vicarious overwork of the tired and irritated kidneys, as also the intestinal fermentation and coprostatic auto-intoxication, which results in some cases.

The author refers to the importance also of examination of the stomach contents after test meals have been given, as these may furnish in some cases a clue to etiologic factors.

Blood examinations he finds quite important in determining the amount of opsonic resistance as also for finding infections in the blood, which matters by lowering the vitality may become factors in the atonic conditions which were being discussed.

---

*Pathology and Diagnosis of Constipation.—By Wm. M. Beach, M. D., of Pittsburgh, Pa.*

Pathology of constipation is naturally considered under two general heads, namely:

1. Stasis due to altered secretions;
2. Stasis due to mechanical obstruction.

The first may be the result of neuroses, and acute fermentative indigestion, or a bacillary infection. The anerobes may attack the contents of the bowel or the gut wall itself, leading to varying degrees of inflammation in the colon,—as ulceration, hypertrophic and atrophic catarrh. The colon impaired functionally or traumatically leads to statis and consecutive inhibition of the fecal excursion. Such impairment further disturbs the physiologic lines of defence against auto-intoxications,—as

(a) The intestinal mucosa, itself;
(b) the liver, and
(c) the antitoxic glands.

Collateral with these phenomena in constipation, are such factors as cholelithiasis, hypochlorhydria, cholangitis and appendicitis, as altered secretions incident to coprostasis.

Mechanical obstrutions to be reckoned with include:

1. Entroptosis or Glenard's disease;
2. Gastroptosis;
3. Dilatation of the colon;
4. Certain extra-mural and intra-mural sources of obstruction,—as pelvic tumors and displacements, nephroptosis, enlarged glands, intussusception, malignant diseases, etc.;
5. Acute angulation at the recto-sigmoid junction, hypertrophy of O'Beirne's spinchter, and stiff rectal valves;
6. Disease in the anal canal.

Diagnosis resolves itself into an analysis of the above conditions; to differentiate acute or chronic obstruction and the ordinary functional stasis which may also be accompanied by the various forms of colitis.

---

*Sequelae of Constipation, Including Auto-Intoxication.—By Alfred J. Zoebel, M. D., of San Francisco, Cal.*

In this paper the writer mentions many of those conditions which seem to have their origin in chronic constipation with auto-intoxication. He states that experimental evidence has not as yet demonstrated that they actually do so, but close observation and clinical experience tend strongly to confirm the theory.

He writes that while all constipated individuals do not necessarily suffer from those symptoms ascribed to auto-intoxication, yet in his experience most patients with auto-toxic symptoms are constipated. This may be without their knowledge, and they often deny in good faith that they are so, but proctoscopic examination generally proves the sigmoid and rectum to be loaded with fecal matter.

A report is given of the proctoscopic observations made on a number of cases of hypertrophic arthritis. In almost every instance the lower bowel was found filled with a fecal mass, although most of the patients positively stated that they had had an evacuation within an hour or two previous to the time of examination. Thorough colonic flushings invariably brought relief from pain, and in time marked improvement in their general condition.

These observations are in line with the theory advanced by various authors that arthritis deformans may be due to intestinal auto-intoxication.

Mention is made of the various muscular, arthritic, and neuralgic pains caused by absorption of toxins from the bowel. These are often misunderstood, and treatment instituted for rheumatism.

Congestion, irritation, and various disturbances, both functional and organic, of the uterus, tubes and ovaries in the female, the vesicles, urethra, and prostate in the male; and the bladder in both, may result from chronic constipation. This is due both to the proximity of these organs to the lower bowel and to their close physiological relationship.

It is noted that albuminuria may arise from intestinal stasis, and mention is made of the opinion advanced by various clinicians that a nephritis may even be caused thereby.

The role of constipation with auto-intoxication as causal factors of epilepsy, neurasthenia, and various mental conditions, as claimed by certain well-known and competent observers, is stated here without comment.

The influence of these conditions on the heart, blood-vessels, and the blood, and its effect on the eye, ear, nose and throat are dilated on in this paper, and in support of these statements quotations are culled from the literature that has appeared on this subject during the past five years.

The writer further briefly mentions a few more of those conditions that are supposed to arise from chronic constipation with auto-intoxication, and concludes by agreeing with the trite observation of Boardman Reed that, "when we except the exanthems, malaria, syphilis, tuberculosis, and the diseases caused by traumatisms, by metallic poisons, and by a few other toxic agents or infections from without, practically all the remaining maladies which afflict us and cut short our lives are now directly or indirectly traceable to auto-intoxication."

---

*Non-Surgical Treatment of Constipation.—By Dwight H. Murray, M. D., of Syracuse, N. Y.*

Dr. Murray stated that chronic constipation and its results was one of the worst of the foes to a healthful human race.

He had never known any medication to cure cases of constipation. As primary causes of constipation he considered CARELESSNESS, IGNORANCE, and LAZINESS to be of first importance. The whole medical profession should teach their clientele how to care for themselves, and to train their children in order that constipation could be eliminated by educational and prophylactic methods.

Medicines for the use of constipated people have increased until their number is almost countless. Advertisements which extol particular cathartics exploited by this or that pharmacist, are well nigh bewildering.

He makes the claim that all cathartics finally leave those who use them worse than before. He does not entirely interdict the use of drugs, as there are cases where they must be used, but almost wholly for temporary relief. He says that a mistaken notion exists in the minds of the laity that the feces is composed largely of debris of food. This, however, furnishes only a comparatively small portion of the fecal mass, the larger portion being deposited in the large intestine as the ash resulting from the products of metabolism.

He mentions various exercises, massage, deep breathing, climbing, rowing, electricity, etc., as being helpful in the treatment and cure of these cases.

Sigmoid injections of pure olive oil, castor oil or medicinal paraffin oil were recommended as aids in the treatment.

He said that hours could be spent over the various drugs and methods in detail—after it all we would be obliged to say, that eternal vigilance as to regularity on the part of the patient must be exercised or a cure would not result.

The keynote of his paper is, education and regularity as to periodicity of the first daily stool. Finally, he believed that the whole profession has a profound duty to perform for mankind in an educational way for emancipating the race from this insidious foe.

---

*The Surgical Treatment of Chronic Constipation.—By Louis J. Hirschman, M. D., of Detroit, Mich.*

Constipation is divided into two great classes, the one class being due to a lack of functional activity, i. e., dietetic error, improper habit, neural or trophic influences. The other class, which some of us have been pleased to designate as obstipation, includes all cases whose impaired activity is due to mechanical interference with the normal peristaltic movements and expulsive function of the bowel.

Obstipation, or obstructive constipation may be caused by;

(1) The presence of any foreign body, occlusion, contracture, hyperhrophy or accumulation in the intestinal canal.

(2) Displacements, acute angulations, distensions, neoplasms, adhesions or compressions of the bowel.

(3) Developmental defects and congenital deviations from normal.

Inasmuch as the surgical treatment of constipation, due to easily recognized local conditions, is obvious, they are dismissed with mere mention. Coloptotic constipation represents such a large percentage of cases of mechanical constipation that its discussion involves the most important field of surgery in the treatment of constipation. All patients with ptotic colons are not constipated, nor do all constipated patients suffer from coloptosis. There must be in addition to ptosis of the cecum, transverse or sigmoidal colons, a condition of functional inactivity due to atony of the bowel muscle.

Suspensions of ptotic colons by means of fixation by adhesions to the abdominal wall are unnatural and interfere with peristalsis. Restoration should be accomplished by shortening the natural support—the mesentery. Lateral anastomoes between the most dependent loops of ptotic bowel is sometimes indicated. Above all, massage, both abdominal and internal rectal, is of primary importance in restoring function, and should be used along with either dietary or hygienic measures to restore bowel function.

---

*Cancer of the Rectum.—By J. Rawson Pennington, M. D., of Chicago, Ill.*

I take it we are all agreed as to the increasing frequency of cancer. At least it seems to me no other conclusion can be drawn from the following figures: According to the 12th U. S. census, cancer appears to have increased 12.1 deaths per 100,000 population in the previous decade. In Great Britain, so we learn from the work of Roger Williams, the deaths from cancer increased from 177 per million in 1840 to 885 per million living in 1905. Williams points out that while the population barely doubled from 1850 to 1905, the mortality from cancer increased more than six fold. Nor is the increase confined to the United States and Europe, it holds good for Japan, India and even for uncivilized countries. In short, cancer is one of the several diseases which is apparently increasing, by leaps and bounds, in spite of our boasted progress in Medicine, Surgery and Hygiene. Apart from the increased prevalence, the present death rate from malignant disease is something dreadful to contemplate. Our anxiety in regard to malignant disease of the rectum is pardonable when we reflect that a good proportion of cancers involve this region. Williams found that 9.6 per cent. in males and 5.3 per cent. in females were located in the rectum. Is there anything that can be done to check this foe? The writer believes there is, and that this Society may be made a powerful factor for good in such a crusade. In Germany a similar crusade has been started against cancer of the uterus by Winters, agitating the subject both among the profession and the laity, it is estimated that the number of cases of inoperable cancer of this organ has been reduced over 30 per cent. as a result of calling attention to the early symptoms. Of the 2,914 cases of rectal cancer in the male referred to by Williams 2,592 patients were over 45 years of age and 2,180 of the 2,533 female patients. In the male sex again the average age, at which the onset was noted, was 49.7 years, the minimum being 16.75 and the maximum 76; while the female sex the average was 50.4 years, with a minimum 21.8 and a maximum of 88 years. This brings me to the crux of my argument, that every person who has reached the so-called "cancerous age" should be examined periodically for evidence of commencing carcinoma, not necessarily of the rectum alone, but in the female, for example, of the uterus also.

In 120 resections of the rectum for malignant disease W. J. Mayo observes: "It is an unfortunate fact that, in the majority, cancer of the rectum is not recognized in time to obtain a radical cure." I said a moment ago that cancer in the beginning is a local disease. This granted, then early and thorough removal must lead to a cure. It has been shown that a large proportion of malignant growths originate in scar tissue. In cancer of the stomach, for example, the Mayos found that no less than 62% showed evidences of a previous ulcer. In rectal cancer patients frequently give a history of previous operations on the part. Does the cancer occur in the scar left from an operation for hemorrhoids done by one of the commoner methods, ligature, clamp and cautery, or some other technic leaving much scar tissue and sometimes stricture? May it not be occasionally engrafted on the

Here is a suggestion for us in our own work, secure smooth healing by resorting only to such procedures as leave the minimum of cicatricial tissue, hence the least possible nidus for possible mischief in the future. With the co-operation of the public it seems to me we should learn much about cancer in the early stages. To educate the public we must—as has been well said—"organize, systematize, deputize, energize, supervise and economize." The field is broad and the opportunity is at hand. Shall we grasp it?

---

*Malformation of Rectum and Anus, with Report of Cases.—By Donly C. Hawley, A. B., M. D., of Burlington, Vt.*

The facts of modern embryology explain a majority but not all developmental defects of the rectum and anus.

M. B., female, age 4 weeks, came under my observation in April, 1910. She had an imperforate anus, the rectum opening into vagina in the upper half of the recto-vaginal septum, opening one-half by one-eighth inch in size, the longer diameter transverse, was evidently supplied with a spinchter, as the child had three or four well controlled movements daily. Anal depression was present and the vulva and vagina were normal, except as noted. The presence of uterus was normal or otherwise not demonstrated. There was no distension of rectum, no impulse and no prominence in perineum. The child was well nourished and otherwise normal. Operative interference postponed. The child is at present well, and is 13 months old and weighs 22 pounds.

While this defect is sometimes seen many cases reported, as atresia ani vaginalis, are no doubt in reality imperforate anal canal with vulvar outlet, a malformation admittedly of common occurrence.

Cases in which intestine opens well up in vagina are not accounted for on embryologic grounds, the two structures being embryologically dissimilar and independent.

*Pruritus Ani, With Report of Cases.—By Donly C. Hawley, A. B., M. D., of Burlington, Vt.*

In this discussion I do not refer to intestinal parsites, errors in diet, etc., in which the pruritis is relieved by proper attention to the causative conditions, nor so much to the symptoms as to the pathologic condition of the skin and nerve endings, which condition is pathognomonic.

The nearly constant local cause of pruritus ani is abrasion and ulceration of the anal canal, accompanied by blind sinuses underneath or fissures in the muco-cutaneous lining.

Further, some cases are associated with chronic protitis, which may be a factor in producing or increasing the anal abrasions or ulcerations.

The treatment I have adopted is as follows:

With the patient wel anaesthetized, the anal canal is dilated, and the ulceration, together with the sinuses and fissures, are thoroughly cauterized with the Paquelin cautery, and also the entire area of chronic dermal inflammation.

My aim is to destroy ulcerated areas, the thickened and altered skin and the pathologic condition of the terminal nerve fibres.

CASE 1. S. H. E., age 62, came under my observation June, 1908. He had suffered with rectal troubles for 45 years. Twenty years ago he was operated on for fissure or fistula—was not certan which. He has had almost intolerable pruritus for eight years, and for the past year it has been so constant and unbearable, especially at night, that he has become a nervous wreck, and has lost 40 pounds in flesh, and has been unable to continue his business.

Diagnosis: Chronic pruritus ani. The skin was inflamed, soddened and thickened over a large area about the anus, with many deep cracks, and four or five ulcerations and abrasions in anal canal.

Treatment as outlined. Result, cure and no return up to present time.

CASE 2. W. A., male, age 38. History of pain in rectum for 20 years, and of severe and intolerable pruritus.

Diagnosis:—Chronic pruritus ani.

There was a large ulceration in anal canal and three or four blind sinuses, with an area of white, brittle and infiltrated skin with large cracks about anus.

Operation, same as in Case No. 1. Result, cure.

Other cases less severe have been operated upon during past three years, with satisfactory results.

The treatment outlined is not new nor original, having been advocated by Mr. W. Mitchell Banks, and practiced by Mr. Fred C. Wallis.

Ball's operation is designed to render anaesthetic the skin over the undercut area.

The operation described accomplishes the same end and besides destroys lesions in anal canal.

The former operation has resulted in extensive sloughing. To the latter no such danger attaches.

(*To Be Continued.*)

---

### GEORGIA STATE TUBERCULOSIS SANATORIUM.

The Executive Committee of the Georgia State Tuberculosis Sanatorium met in Atlanta on the eleventh inst. There were present Dr. T. R. Whitley, Pres.; Dr. Jeff Davis, Chairman; Dr. H. R. Slack, Vice-Chairman; Dr. C. H. Richardson, and Miss Rosa Lowe, Sec.

The resignation of Dr. E. W, Glidden, the present superintendent, was accepted and after carefully considering the list of applicants for the position, Dr. Wm. V. Parramore was unanimously elected superintendent.

Dh. Parramore is a native Georgian, a graduate of the Uuiversity of Maryland and is now assistant superintendent of the Maryland Tuberculosis Sanatorium. He comes to the Georgia Sanatorium very highly endorsed.

The sanatorium is now full, but the trustees will have to charge $3.50 per week for seventy-five per cent. of the patients after November the first, as the Legislature did not appropriate sufficient funds to carry on the work as required, and it is best to make this small charge so as to care for all who can be accommodated.

